# Psychometric properties of a patient‐reported outcome set in acute stroke patients

**DOI:** 10.1002/brb3.2249

**Published:** 2021-06-14

**Authors:** Rebecca Philipp, Lisa Lebherz, Götz Thomalla, Martin Härter, Hannes Appelbohm, Marc Frese, Levente Kriston

**Affiliations:** ^1^ Department of Medical Psychology University Medical Center Hamburg‐Eppendorf Hamburg Germany; ^2^ Department of Neurology University Medical Center Hamburg‐Eppendorf Hamburg Germany; ^3^ Office for Quality Management and Clinical Process Management University Medical Center Hamburg‐Eppendorf Hamburg Germany

**Keywords:** ICHOM, patient‐reported outcome measures, PROMIS, psychometric properties, stroke, validation

## Abstract

**Objectives:**

Impairments after stroke may affect multiple domains of health‐related quality of life (HRQoL). Patient‐reported outcome measures (PROMs) have proven valuable in measuring patients’ well‐being. We examine the psychometric properties of a standard set of PROMs assessing global health, anxiety, and depression, and functioning in a German health care setting.

**Method:**

We included inpatients at the Department of Neurology at the University Medical Center Hamburg‐Eppendorf, diagnosed with stroke. Following the stroke‐specific standard set of the International Consortium for Health Outcome Measurement, we collected demographic and clinical information at baseline, and PROMs for global health (PROMIS‐10), three items for self‐reported functioning, anxiety, and depression (PHQ‐4) at 90 days follow‐up. We calculated confirmatory factor analyses to test factorial validity and correlation analyses to test construct validity. We further conducted item and reliability analyses.

**Results:**

In a sample of 487 patients (mean age, SD: 71.1, 12.6; 47% female) with mild and moderate symptoms, model fit for the PROMIS‐10 was acceptable for the two‐factor and single‐factor models. Factor loadings ranged from 0.52 to 0.94. The postulated single‐factor model for functioning was saturated with zero degrees of freedom. Factor loadings ranged from 0.90 to 0.96. For the PHQ‐4, the two‐factor model showed excellent model fit. Factor loadings ranged from 0.78 to 0.87. Internal consistency was acceptable to good. Construct validity was generally confirmed.

**Conclusions:**

The PROMIS‐10 is a valid and reliable instrument to measure HRQoL among German stroke patients. While the PHQ‐4 was confirmed as a screening measure for mental disorders, further research is needed on items assessing self‐reported functioning. Results are limited to patients showing minimal functional deficits.

## Graphical abstract

We examine the psychometric properties of a standard set of PROMs assessing global health, anxiety and depression, and functioning in a German health care setting. The PROMIS‐10 is a valid and reliable instrument to measure HRQoL among German stroke patients. While the PHQ‐4 was confirmed as a screening measure for mental disorders, further research is needed on items assessing self‐reported functioning. Results are limited to patients showing minimal functional deficits.

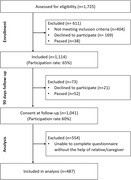

## 1 INTRODUCTION

To improve patients’ health‐related quality of life (HRQoL), patient‐reported outcome measures (PROMs) have a strong impact on decisions made in the context of evaluating patient care (Reeves et al., 2018; Snyder et al., 2013; Valderas & Alonso, 2008). Next to physiological and other medical information, patients’ health status is now also assessed by their subjective experiences with health‐related domains such as mental well‐being, functional impairment, psychosocial functioning, and quality of life (Glasgow et al., 2012; Ishaque et al., 2019; Willke et al., 2004). Although the benefits and costs of implementing PROMs into routine care have been critically discussed (Gilbody et al., 2002; Greenhalgh et al., 2005; Marshall et al., 2006), assessing PROMs has a positive impact on patient satisfaction, process of care, and health outcomes (Ishaque et al., 2019; Recinos et al., 2017), further promoting the shift toward an increased patient‐centeredness of medical care (Baumhauer, 2017; Glasgow et al., 2012).

Aiming to coordinate and to standardize the rising number of PROMs (Kotronoulas et al., 2014; Price‐Haywood et al., 2019), the National Institute of Health (NIH) established the Patient‐Reported Outcomes Measurement Information System (PROMIS) (Cella et al., 2010). Among the disease‐specific standard sets, published by the International Consortium for Health Outcome Measurement (ICHOM), is the Standard Set for Stroke (ICHOM‐SSS), which was developed qualitatively based on expert consensus, with the primary aim of creating a clinically intuitive and practical measure (Salinas et al., 2016). Independent from the severity of stroke symptoms, the impairments that may occur after stroke have the potential to affect every health‐related domain including HRQoL (Katzan, Schuster, et al., 2018; Katzan, Thompson, et al., 2018; Katzan et al., 2019; Price‐Haywood et al., 2019). Thus, PROMs are a valuable addition to well‐established clinician‐reported measures in order to capture changes relevant to the patients’ well‐being (Katzan et al., 2017).

In the ICHOM‐SSS, one of the measures to assess patient‐reported health status is the PROMIS Global Health short form (PROMIS‐10). The instrument measures the patients’ global health status based on a global physical health score (GPH) and a global mental health score (GMH). Both scales have been validated in previous studies: Hays and colleagues (2009) suggested a two‐factor structure (GPH, GMH) with four items each, after rejecting a single‐factor solution in a confirmatory factor analysis (CFA). Katzan and Lapin (2018) were also able to confirm the suggested two‐factor solution. Although results of the CFAs in both studies showed acceptable model fit, their models excluded two single items and the authors identified inconsistencies with global goodness‐of‐fit indices. Because the PROMIS‐10 is measured using 10 items, a validation study should take into account all items and address these shortcomings.

In addition to patients’ global health status, their functional impairment and potential mental disorders are considered central patient‐reported outcomes (Poku et al., 2016; Price‐Haywood et al., 2019). Both are strongly associated with HRQoL after stroke (Rafsten et al., 2018; Tramonti et al., 2014; Wilson & Cleary, 1995). However, functional impairment after stroke is usually assessed by clinicians evaluating the patients’ physical and cognitive impairments (Harvey, 2015; Jönsson et al., 2014; Lyden et al., 1994) neglecting the patient perspective. As for mental disorders, systematic reviews and meta‐analyses on post‐stroke anxiety (PSA) and post‐stroke depression (PSD) report prevalences between 29% and 31% (Ayerbe et al., 2013; Hackett & Pickles, 2014; Rafsten et al., 2018). In the included studies, PSA and PSD were mostly diagnosed using self‐report measures (e.g., Hospital Anxiety and Depression Scale, Hamilton Anxiety Rating Scale, Generalized Anxiety Disorder 7‐item scale, Beck Depression Inventory, and Patient Health Questionnaire). Another meta‐analysis on PSD, in which patients were diagnosed based on clinical interviews, prevalences ranged between 11% and 18% (Mitchell et al., 2017). Since mental disorders may affect recovery and rehabilitation after stroke (Belagaje, 2017; Nannetti et al., 2005), these findings stress the need for reliable and valid patient‐reported outcomes to assess symptoms of anxiety and depression after stroke.

In the context of a larger study implementing the ICHOM‐SSS in routine stroke care in Germany (Rimmele et al., 2019), we aimed to test the psychometric properties of a patient‐reported outcome set in patients with stroke 90 days after a cerebrovascular incident to further confirm the factor structure of the PROMIS‐10 and its validity in a German‐speaking sample. First, we tested the factorial validity of the PROMs. Therefore, we aimed to confirm (a) the two‐factor structure of the PROMIS‐10 measuring global health (Hays et al., 2009; Katzan & Lapin, 2018); (b) a heuristically postulated single‐factor structure of the ICHOM‐SSS items for functional impairment (self‐reported functioning) (Salinas et al., 2016); (c) the two‐factor structure of the Patient Health Questionnaire‐4 (PHQ‐4) measuring anxiety and depression to test its validity to screen for these symptoms in patients with stroke (Kroenke et al., 2009). Second, we aimed to determine construct and discriminant validity. Therefore, we expected (a) GPH to show stronger negative associations with self‐reported and clinician‐rated functioning than with anxiety and depression; (b) GMH to show stronger negative associations with anxiety and depression than with self‐reported and clinician‐rated functioning; (c) substantial correlation between self‐reported and clinician‐rated functioning; and (d) a moderate positive association between GPH and GMH.

## 2 METHODS

### 2.1 Study design and study sample

This psychometric study is part of a prospective exploratory observational and implementation study, which is currently conducted at the Department of Neurology at the University Medical Center Hamburg‐Eppendorf, Germany. The hospital's stroke unit cares for all regular patients with stroke admitted to the hospital. There, we consecutively recruited inpatients who were diagnosed with acute ischemic or hemorrhagic stroke over a period of 15 months. We excluded patients who showed severe deficits in their ability to communicate (e.g., dementia or aphasia). All patients or their legal guardians provided informed consent. Please see the study protocol for more detailed information and primary research questions (Rimmele et al., 2019). The study protocol was approved by the ethics committee of the Hamburg chamber of physicians. The study is registered at clinicaltrials.gov, NCT03795948.

Following the ICHOM‐SSS (Salinas et al., 2016), there were four points of assessment: baseline (admission to the hospital), discharge from the hospital, and 90‐day and 12‐month follow‐up. For this study, we used the information collected at baseline (demographic, diagnostic and clinical information, functional impairment, and patient‐reported health prior to the stroke) and at the 90‐day follow‐up (patient‐reported outcomes including functional impairment). At baseline, study participants completed the paper–pencil version of the ICHOM‐SSS during their hospital stay. If they were unable to complete it by themselves, a research assistant administered the items in an in‐person interview. Follow‐up questionnaires were sent to the patients after discharge. In case the patients indicated need for assistance, items were administered in a telephone interview. Patients' ability to complete the questionnaire without help was assessed by a separate item.

### 2.2 Measures

We collected basic demographic and clinical characteristics from the patients’ electronic health record. We assessed all other information according to the German version of the ICHOM‐SSS (Supporting information S1). This included details on the *stroke event*, such as prior vascular and systemic diseases, risk factors, stroke severity, duration of symptoms (less than 1 hour, 1 hour to 1 day, longer than 1 day, unable to determine), and level of consciousness at arrival (fully awake, somnolent, coma). Stroke severity was measured using the NIH Stroke Scale (Lyden et al., 1994), with scores of 0 indicating no stroke symptoms, 1–4 mild, 5–15 moderate, 16–20 moderate to severe, and 21–42 severe stroke symptoms. We assessed the patient‐reported health status using the PROMIS‐10 and the three ICHOM‐SSS items regarding patients’ functional impairment. Moreover, we assessed symptoms of anxiety and depression using the PHQ‐4 (Kroenke et al., 2009), a brief self‐report questionnaire not part of the ICHOM‐SSS.

The *PROMIS‐10* measures the patients’ global health status based on a GPH, which includes the domains physical health, mobility, pain, and fatigue, and a GMH, which includes the domains quality of life, mental health, satisfaction with social activities, and mood. The remaining two items (general health, social participation) of the 10‐item instrument are scored separately. Patients were asked to answer the questions regarding their HRQoL in the past 7 days on a five‐point Likert scale from 1 (poor or not at all) to 5 (excellent or completely). Higher values indicate higher HRQoL. To calculate sum scores, the items for mood (always to never) and fatigue (none to very severe) are reversely coded, and the item for pain ranging from 1 (no pain) to 10 (worst imaginable pain) is recoded into five categories (no, mild, moderate, severe, and very severe pain). The sum scores for the GPH and the GMH with four items each range from 4 to 20. Both sum scores were converted into *T*‐scores (standardized to have a population mean of 50 and a population standard deviation of 10 points), according to the ICHOM Stroke Data collection reference guide (International Consortium for Health Outcomes Measurement, 2018).

For *self‐reported functional impairment* as assessed by the ICHOM‐SSS, patients were asked to indicate whether they needed help walking, going to the toilet, and getting dressed. Patients had three response options for the first item (e.g., 1 = able to walk without help, 2 = able to walk with help, 3 = unable to walk) and two response options for the latter two items. For validation purposes, we used the clinician‐rated functional impairment as assessed by the simplified modified Ranking Scale questionnaire (smRSq; Bruno et al., 2013; van Swieten et al., 1988), which measures patients’ degree of disability or dependence. The scale consists of one item, which is scored on a seven‐point Likert‐scale ranging from 0 (no symptoms) to 6 (death) and was assessed via a telephone assessment 90 days after stroke with either the patient or a patient's relative or care taker.

*Anxiety and depression* symptoms were assessed using the German version of the PHQ‐4 (Kroenke et al., 2009), in which both subscales are represented by two items each. Patients were asked to indicate on a four‐point Likert scale ranging from 0 (not at all) to 3 (nearly every day) how often they experienced symptoms of these disorders over the last 2 weeks. Accordingly, the sum scores of both subscales and the total scale range from 0 to 6 and 0 to 12, respectively. Higher values indicate more anxiety and depressive symptoms. The questionnaire's two‐factor structure was validated and is preferable to the one‐factor model (Löwe et al., 2010).

### 2.3 Statistical analyses

We calculated descriptive statistics for sample characteristics (frequencies, means, and SDs), and performed item analysis for the patient‐reported outcome measures (means, SDs, skewness, and kurtosis). To assess reliability, we calculated Cronbach's *α* as a measure of internal consistency for each scale as well as the standardized difficulty and corrected item‐total correlation for each item.

We performed our analyses within the framework of classical test theory. It should be noticed that several other approaches exist, most prominently item‐response theory, which may model the response pattern in the data even better than the methods applied here. We conducted a series of analyses to test the factorial structure of the investigated measures. First, we tested the hypothesized two‐factor structure of the PROMIS‐10 with four items loading on a global mental health factor (GMH), and four items loading on a global physical health factor (GPH). We allowed the two single items global health (global01) and social participation (global09) to be correlated with both factors. Second, we tested a single‐factor model to examine whether the three categorical items for self‐reported functioning poststroke were loading on one latent factor. Third, we aimed to confirm the two‐factor structure of the PHQ‐4 with the items measuring nervousness and worries loading on the anxiety factor, and the items measuring loss of interest and depressive mood loading on the depression factor (Löwe et al., 2010). In all confirmatory factor analyses, we evaluated model fit based on the following indices (Hu & Bentler, 1999; Kriston et al., 2008; Schermelleh‐Engel et al., 2003): normed *χ* ^2^ (*χ* ^2^/degrees of freedom (df) < 3.0 for good, < 5.0 for acceptable fit), comparative fit index (CFI > 0.95 for good, > 0.90 for acceptable fit), Tucker–Lewis index (TLI > 0.95 for good, > 0.90 for acceptable fit), root means error of approximation (RMSEA < 0.05 for good, < 0.08 for acceptable fit), standardized root mean squared residual (SRMR < 0.05 for good, < 0.08 for acceptable fit), and weighted root mean square residual for weighted least square mean and variance adjusted estimation used in the CFA with categorical variables (WRMR < 1.0 for good fit; DiStefano et al., 2017). We report standardized factor loadings, which can be interpreted as the strength of association between the observed items and the latent factor or the quality, how well the item measures the factor. Usually, standardized factor loadings of 0.40 or higher are interpreted as acceptable. We also report the results of the *χ* ^2^ tests for completeness and the Bayesian information criterion (BIC) for model comparisons. For both, lower values indicate less discrepancy between data and theory, that is, a better model fit. We examined modification indices (MI) post hoc for identifying ways of how the model can be improved further. MI are an estimate of the amount by which *χ* ^2^ can be reduced if a parameter restriction was removed, for example, if a residual correlation is estimated instead of being restricted to 0.

To test construct validity of the scale scores resulting from the measures, we calculated Pearson's correlation coefficients between the two scales of the PROMIS‐10 (GPH, GMH), self‐reported and clinician‐rated functioning, as well as the subscales of the PHQ‐4 (Cohen, 1992). We calculated the correlation between GPH and GMH to test discriminant validity.

We considered findings with *p* < .05 as statistically significant. We used IBM SPSS Statistics, Version 25 (IBM Corp., 2017) for descriptive and reliability analyses. We conducted confirmatory factor analyses with Mplus, Version 7.1170 (Muthén & Muthén, 1998–2010).

## 3 RESULTS

### 3.1 Study sample

**FIGURE 1:**
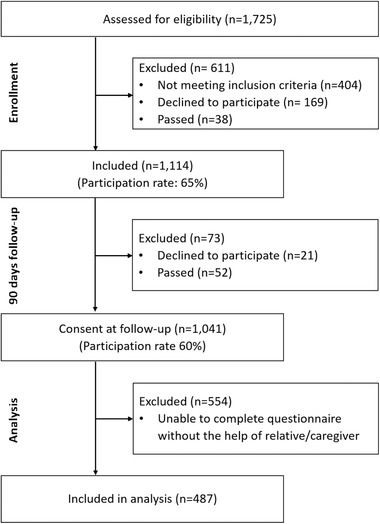
Flow diagram

We collected data of 1,725 patients between March 2017 and June 2018. This sample comprised all patients admitted to the stroke unit. In this psychometric study, we excluded patients who did not give consent at the 90‐days follow‐up (*n* = 684) and who were unable to complete the questionnaire without the help of a relative or caregiver (*n* = 554) due to possible bias. The flow diagram describes participation in detail (Figure 1). Table 1 shows detailed demographic and clinical characteristics of the sample. The final sample of 487 patients consisted almost equally of men and women. About two‐thirds were diagnosed with cerebral ischemia, about one‐third presented with a transient ischemic attack or amaurosis fugax/retinal artery occlusion, and only 4% suffered from an intracerebral hemorrhage. A large proportion (82%) of the sample suffered from no or minor stroke symptoms. Most of the patients had had a stroke prior to the current cerebrovascular incident.

**TABLE 1:** Demographic and clinical sample characteristics (N = 487)

Variable	N	%
Age, mean (SD, range)	71.12 (12.59, 22–97)	
Gender		
Female	228	47
Male	259	53
Type of stroke		
Cerebral ischemia	298	61
Transient ischemic attack	116	24
Amaurosis fugax or retinal artery occlusion	53	11
Intracerebral hemorrhage	19	4
Stroke severity^†^		
No stroke symptoms	196	40
Mild stroke symptoms	202	42
Moderate stroke symptoms	81	17
Moderate to severe stroke symptoms	6	1
Severe stroke symptoms	1	0.2
Clinician‐rated functional status^‡^, mean (SD, range)	0.83 (0.87, 0–4)	
Duration of symptoms		
< 1 hour	79	16
1 hour to 1 day	296	61
> 1 day	94	19
Unable to determine	17	4
Level of consciousness at arrival		
Fully awake	475	98
Somnolent	11	2
Prior vascular diseases		
Stroke	78	84
TIA	17	4
Myocardial infarction	33	7
Coronary artery disease	55	11
Atrial fibrillation	70	15
Prior systemic diseases		
Diabetes	56	12
Hypertension	292	60
Hyperlipidemia	71	15
Risk factors		
Smoking	100	22
Alcohol (more than one beverage per day)	37	8

^†^ As assessed by the NIH Stroke Scale (Lyden et al., 1994).

^‡^ As assessed by the simplified modified Ranking Scale questionnaire (Bruno et al., 2013; van Swieten et al., 1988).

### 3.2 Factorial validity

**FIGURE 2:**
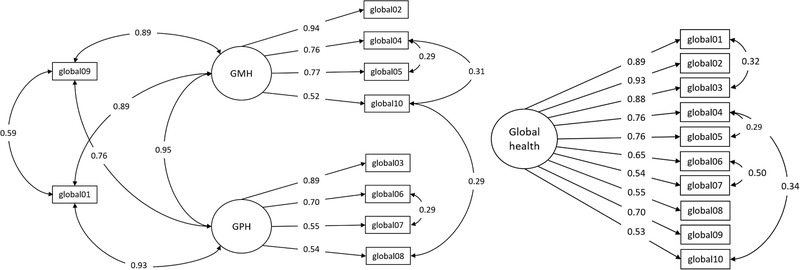
Standardized parameter estimates for the two‐ and single‐factor models with residual correlations, without error terms. GMH = global mental health, GPH = global physical health, Global01 = general health status, global02 = health‐related quality of life, global03 = physical health, global04 = global cognitive function, global05 = satisfaction with social activities, global06 = mobility, global07 = pain, global08 = fatigue, global09 = social participation, global10 = emotional problems

Standardized factor loadings for the suggested two‐factor model of the PROMIS‐10 ranged between 0.55 and 0.93. They were lowest for the three recoded items. The model showed poor fit for all indices, except the SRMR (Table 2). After we examined the MI, to identify how the model can be improved by reducing parameter restrictions, we added residual correlations for the following items: global cognitive functioning (global04) and satisfaction with social activities (global05) (MI = 39.13), global cognitive functioning and emotional problems (global10) (MI = 62.23), emotional problems and fatigue (global08) (MI = 49.65), and mobility (global06) and pain (global07) (MI = 37.57). Standardized factor loadings for this adapted model ranged between 0.52 and 0.94. Model fit improved (Table 2) and was acceptable, except for the normed *χ* ^2^ and RMSEA. The BIC confirmed that this model (Figure 2) fit the data better than the model without residual correlations.

**TABLE 2:** Global fit indices for the tested factor models of the PROMIS‐10

		Global fit indices						
Model	*χ*^2^ test	Degrees of freedom	Normed *χ* ^2^	CFI	TLI	RMSEA	SRMR	BIC
Two‐factor model without residual correlations	409.85, *p* < .001	31	13.22	0.89	0.84	0.16	0.06	10,261.70
Two‐factor model with residual correlations	223.89, *p* < .001	27	8.29	0.94	0.91	0.12	0.04	10,100.49
Single‐factor model without residual correlations	503.40, *p* < .001	35	14.38	0.87	0.83	0.17	0.07	10,330.50
Single‐factor model with residual correlations	225.95, *p* < .001	31	7.29	0.94	0.92	0.11	0.05	10,077.80

Abbreviations: BIC, Bayesian information criterion; CFI, comparative fit index; Normed χ^2 ^, χ^2^/degrees of freedom; RMSEA, root means error of approximation; SRMR, standardized root mean squared residual; TLI, Tucker–Lewis index.

As both latent factors were highly correlated (*r* = 0.95), we also tested a model with a single global health factor in a post hoc analysis, which fit our data poorly regarding all indices (Table 2). To improve model fit, we added residual correlations between the items for mobility and social participation (global09) (MI = 114.84), global cognitive functioning and emotional problems (MI = 68.46), general health status (global01) and physical health (global01) (MI = 60.31), and global cognitive functioning and satisfaction with social activities (MI = 56.37). Standardized factor loadings for the adapted single‐factor model ranged between 0.53 and 0.93 (Figure 2). Model fit improved (Table 2) and was acceptable, except for the RMSEA.

For self‐reported functioning (functional impairment), standardized factor loadings in the single‐factor model were 0.91 for ambulation, 0.96 for toileting, and 0.90 for getting dressed (Figure S1). Due to the limited number of indicators, the model was saturated with zero degrees of freedom. This means that the number of parameters that had to be estimated was equal to the amount of information available in the observed data and therefore global model fit could not be assessed.

Further, we confirmed the two‐factor structure of the PHQ‐4. Model fit was excellent (*χ* ^2^ = 0.22, *p* = .64, df = 1, *χ* ^2^/df = 0.22; CFI = 1.00; TLI = 1.01; RMSEA < 0.001; SRMR = 0.002) and standardized factor loadings ranged between 0.78 and 0.89 (Figure S1). The latent factors anxiety and depression correlated highly with *r* = 0.98.

### 3.3 Construct validity

We calculated Pearson's correlation coefficients between the constructs of interest to test discriminant and construct validity (Table 3). Due to the sample size, results should be interpreted based on the strength of the associations as indicated by the correlation coefficient rather than the *p*‐values. The global health scales of the PROMIS‐10 correlated strongly that indicates that the factors are largely overlapping and cannot be easily differentiated, suggesting limited discriminant validity. The negative associations between GMH and anxiety and depression were stronger than those between GMH and with self‐reported or clinician‐rated functional impairment indicating construct validity for this subscale. The negative associations between GPH and anxiety and depression were at least as strong as for self‐reported or clinician‐rated functional impairment. This finding is not fully consistent with theoretical expectations and indicates limited construct validity.

**TABLE 3:** Bivariate correlation coefficients for global physical health, global mental health, and external criteria 90 days after stroke (N = 487)

	GPH	GMH	Depression	Anxiety	Self‐rated functioning
GMH	0.73				
Depression	–0.59	–0.72			
Anxiety	–0.50	–0.66	0.77		
Self‐reported functioning^†^	–0.31	–0.26	0.23	0.24	
Clinician‐rated functioning^‡^	–0.53	–0.41	0.30	0.25	0.40

*Notes*. All correlations were statistically significant with *p* < .001.

Abbreviations: GMH, global mental health; GPH, global physical health.

^†^ Functional impairment as assessed by the ICHOM‐SSS items for functional impairment: ambulation, toileting, and dressing.

^‡^ Functional impairment as assessed by the simplified modified Ranking Scale questionnaire (Bruno et al., 2013; van Swieten et al., 1988).

### 3.4 Reliability

Acceptance of the three patient‐reported outcomes was high with data missing in three cases at most.

For the PROMIS‐10, patients in this sample experienced good to very good global health with item means ranging from 2.85 (physical health) to 4.16 (mobility). Visual examination of the histograms as well as skewness and kurtosis values showed that the items were approximately normally distributed (Table 4). Corrected item‐total correlations ranged between 0.50 (fatigue) and 0.80 (global cognitive functioning). Cronbach's alpha coefficients for both scales indicate acceptable (GPH) to good (GMH) internal consistency.

**TABLE 4:** Item and scale characteristics of the German version of the PROMIS‐10

			Item characteristics						
Item	Content	Cronbach's *α*	N	Mean (*T*‐score)	Standard deviation	Standar‐dized difficulty	Corrected item‐total correlation	Skewness	Kurtosis
**Global physical health (GPH)**		0.79		14.51 (46.3)	2.99				
global03	Physical health		484	2.85	0.85	0.46	0.66	0.56	0.17
global06	Mobility: Everyday physical activities		485	4.16	1.03	0.79	0.64	–1.05	0.29
global07^†^	Pain		482	4.07	0.90	0.77	0.59	–0.62	–0.55
global08^†^	Fatigue		485	3.42	1.03	0.61	0.50	0.06	–0.83
**Global mental health (GMH)**		**0.86**		13.03 (45.8)	3.18				
global02	Health‐related quality of life		485	3.05	0.91	0.51	0.74	0.33	–0.11
global04	Global cognitive function		486	3.13	0.96	0.53	0.80	0.10	–0.26
global05	Satisfaction with social activities		485	3.28	0.92	0.57	0.75	0.12	–0.30
global10	Emotional problems		486	3.57	0.99	0.64	0.58	–0.24	–0.65
**Standalone items**									
global01	General health status		485	2.97	0.89	0.49	n. a.	0.37	0.06
global09	Social participation		485	4.02	0.94	0.76	n. a.	–0.74	–0.01

Abbreviation: n.a., not applicable.

^†^ Recoded items.

^‡^ Cronbach's *α* is not defined for the standalone items.

Patients reported higher mental than physical global health, yet it is unlikely that the mean difference between the two scales was clinically relevant. According to the scoring guide provided by the ICHOM (ichom.org/files/medical‐conditions/stroke/stroke‐reference‐guide.pdf, accessed April 26, 2020), raw scores for GPH and GMH in this sample correspond with *T*‐scores (*M* = 50, SD = 10) of *M* = 46.3 and *M* = 45.8, respectively.

More than 95% of the patients reported high functioning as they were able to walk, go to the toilet, and dress themselves without help from another person. Cronbach's alpha for the functioning scale indicated acceptable internal consistency. Table S1 shows a summary of the reliability analyses.

The majority of patients did not report any symptoms of anxiety or depression with item means ranging from 0.47 (worries) to 0.69 (loss of interest). Cronbach's alpha coefficients for both scales indicate acceptable (anxiety) to good (depression) internal consistency. Table S2 shows a summary of the reliability analyses.

## 4 DISCUSSION

In this psychometric study, we examined the properties of a patient‐reported outcome set consisting of a measure of health‐related quality of life (PROMIS‐10), self‐reported functioning (three corresponding items of the ICHOM‐SSS), and symptoms of depression and anxiety (PHQ‐4), in a German sample of stroke patients.

We aimed to validate the previously suggested two‐factor structure of the PROMIS‐10 (Hays et al., 2009; Katzan & Lapin, 2018) in a German sample testing a model that included general health status and social participation as stand‐alone items. Other than hypothesized, we found that the model with a global physical and a global mental health factor was not preferable to a model with a single global health factor in our data. The high correlation between GPH and GMH suggests low discriminant validity for both factors, indicating that the physical and mental health experienced by the patients in our sample may be considered as single global health factor. This may be due to the fact that most of the patients suffered only from mild motor or cognitive symptoms. Their physical symptom burden may have been strong enough to cause impairments in everyday life, such as poor social functioning, and, thus, affected patients’ mental health. At the same time, the physical symptom burden may not have been severe enough to have patients focusing as much on their physical as on their mental well‐being. Due to the poor fit of the two‐factor model, Katzan and Lapin (2018) concluded to use the single items to describe HRQoL in stroke patients. While our results are consistent with their findings, the advantages of using single items compared to using the two‐ or single‐factor solutions need to be studied further. In addition to the empirical data, this discussion should include reflections about the clinical relevance of the different approaches.

The two‐ and single‐factor models showed good model fit after adding residual correlations post hoc based on the results of the CFAs. The varying factor loadings and correlated errors, if confirmed by independent studies, may suggest forming weighted scores for both subscales. The correlations between the items assessing mood, general health and physical activity, and emotional problems and fatigue were also reported by Hays et al. (2009). However, as the residual correlations were low and could be explained by the item contents, they are unlikely to raise serious concerns during application of the measure. Nonetheless, they seem to be present in different settings, therefore they deserve further attention in independent psychometric investigations. Also, the RMSEA, a standardized measure of the amount of the error in the model, was above the frequently used threshold of 0.08 and, thus, did not always support the tested models. However, the RMSEA tends to be inflated in models with few strongly correlated variables (Kenny et al., 2014; Kenny & McCoach, 2003; Shi et al., 2019). Therefore, we think in this situation the other fit indices should have more weight.

According to the *T*‐scores, our sample reported lower GPH and GMH than the general US population (ichom.org/files/medical‐conditions/stroke/stroke‐reference‐guide.pdf, accessed April 26, 2020; Hays et al., 2009). Means for both scales were similar when compared to the North American stroke sample studied by Katzan and Lapin (2018). Even though those patients and the patients in our study appear to have experienced a similar extent of disability after stroke, patients in our sample reported slightly higher GPH. This may be explained by the fact that the patients in the present study were 10 years older on average. It is possible that older patients have already had more time to adapt to the age‐related decline in physical health resulting in less additional impairment due to mild stroke symptoms. This assumption is supported by the “Gender and Age Range Sub‐norms for Adult PROMIS Measures Centered on the US General Census 2000,” reported on http://www.healthmeasures.net/score‐and‐interpret/interpret‐scores/promis/reference‐populations (accessed April 26, 2020). The subnorms for adults between 65 and 74 years were *M* = 51.0 (SD = 8.8) for GPH and *M* = 53.1 (SD = 9.9) for GMH, as compared to adults between 18 and 34 years with *M* = 51.6 (SD = 8.4) for GPH and *M* = 48.5 (SD = 9.7) for GMH.

Construct validity of the PROMIS‐10 could generally be confirmed by strong negative associations with related measures of functioning and symptoms of anxiety and depression. The limited discriminant validity of the two dimensions of the PROMIS‐10 was also reflected by the fairly comparable correlations of the domain scores with the external measures.

We tested a reflective, saturated model for self‐reported functioning with high factor loadings for all three items. However, we heuristically decided on a single‐factor structure corresponding with the three items suggested by the ICHOM‐SSS. To measure self‐reported functioning more comprehensively, future validation studies may benefit from models that include more indicators, which assess functioning in a more differentiated manner, such as the PROMIS‐Physical Function item bank (Rose et al., 2014), the Stroke Impact Scale‐16 Scale (Duncan et al., 2003), and the Stroke Specific Quality of Life Scale (Ewert & Stucki, 2007). The moderate associations between self‐reported functioning and the external measures suggest low construct validity. Especially the association between self‐reported and clinician‐rated functioning was weaker than expected. Since both constructs aim to assess the patients’ disability or dependence, one possible interpretation may be that the clinicians’ and patients’ perspectives differ to a substantial extent, which is a common finding in the care of stroke patients (Katzan et al., 2017; Price‐Haywood et al., 2019). This underlines the importance of assessing self‐reported functioning using adequate measures.

We were able to confirm the two‐factor structure of the PHQ‐4 showing that the questionnaire is a reliable and valid measure to screen for symptoms of anxiety and depression in patients with stroke. Yet, the correlation between both factors was high indicating low discriminant validity, which is further supported by the results of the correlation analysis. Still, construct validity of the PHQ‐4 was satisfactory, as symptoms of anxiety and depression showed weaker associations with GPH than with GMH and were only moderately associated with both measures of functioning. Other than suggested by the cited references, patients screening positive for symptoms of depression (11% with a score ≥3) and anxiety (12% with a score ≥3) were underrepresented in our sample, which may be due to their higher functioning.

There are limitations to our study. Our findings cannot be generalized to patients with severe symptoms because only few of the patients in our sample suffered from moderate to severe stroke symptoms. Since this was also the case for the earlier study by Katzan and Lapin (2018), there is currently no evidence for the use of the PROMIS‐10 among more severely impaired patients. At the same time, we excluded those patients from the psychometric study who did not complete the questionnaire by themselves but had a relative or caregiver completing it for them in order to control for potential bias (the administered measures were designed to be answered by the patients). However, it is likely that this limits the generalizability of our findings because patients who suffer from more severe stroke symptoms do not have the mental or physical capacity or find it too distressing to fill out the questionnaire. Accordingly, patient‐reported outcomes like the PROMIS‐10 may only apply to patients who show mild or moderate impairment (George & Zhao, 2018). It is possible that our findings are limited due to a skewed distribution of data, especially for the self‐reported functioning and the PHQ‐4, which suggests floor effects. This may be explained by the overall low distress reported by patients in our sample. With regard to generalization, it is also notable that we recruited patients from a university medical center. In case of a cerebrovascular incident, patients might be attended to more quickly in this specific, urban clinical setting than in rural areas. Moreover, we were unable to determine construct and discriminant validity of the PROMIS‐10 in a narrower sense, because the ICHOM‐SSS does not include similar and distinct constructs. Since this psychometric study was part of a larger clinical study testing the implementation of the ICHOM‐SSS, we used the items provided by the standard set and added the PHQ‐4 to maintain efficiency and keep the possible burden for patients at a minimum.

## 5 CONCLUSION AND IMPLICATIONS

Although there have been previous attempts to conceptualize the rising number of available PROMs (Valderas & Alonso, 2008), the ICHOM has been essential in providing standardized, efficient, and disease‐specific set of PROMs. The PROMIS‐10 of the ICHOM‐SSS provides a standard set of items for assessing the HRQoL in stroke patients. We were able to show that the German version of the PROMIS‐10 is a valid and reliable instrument to measure HRQoL among stroke patients with mild to moderate symptoms. Our findings are in line with previous validation studies on the structure of the PROMIS‐10 (Hays et al., 2009; Katzan & Lapin, 2018). Yet, the psychometric limitations also found in our study suggest that there may be alternative approaches to measure global HRQoL. In addition to the PROMIS‐10, we were able to show the value of measuring self‐reported functioning and symptoms of anxiety and depression in this population to further integrate the patient perspective into routine care. While the PHQ‐4 has proven to be a valid and reliable instrument to screen for mental disorders, our study offers new information on the assessment of self‐reported functioning. PROMs measuring functional impairment need to explore aspects that patients find most relevant to their functioning and assess them using a comprehensive item pool. In addition, an in‐depth investigation of the level and conditions of agreement between self‐report and clinician assessment of functional impairment is urgently needed. Future research is needed to address the practicability and benefit of the PROMIS‐10 and self‐reported functioning, especially among patients who suffer from moderate to severe symptoms.

## FUNDING INFORMATION

Innovation Fund of the German Federal Joint Committee, grant number: 01VSF16023.

## CONFLICT OF INTEREST

GT reports receiving consulting fees from Acandis and Portola, grant support and lecture fees from Bayer, lecture fees from Boehringer Ingelheim, Bristol‐Myers Squibb/Pfizer, and Daiichi Sankyo, and consulting fees and lecture fees from Stryker, all outside this work. All other authors declare no conflict of interest.

### PEER REVIEW

The peer review history for this article is available at https://publons.com/publon/10.1002/brb3.2249.

## Supporting information



Supporting Information

## Data Availability

Participant data and other material will not be available.
