# Supplementary material for: Psychometric properties of a patient‐reported outcome set in acute stroke patients
Source: Brain Behav. 2021 Jun 14;11(8):e2249. doi: 10.1002/brb3.2249 (PMC8413767; doi:10.1002/brb3.2249)
Supplement: Supplementary file 1 — Supporting Information [file BRB3-11-e2249-s001.docx]

S1. Availability of the patient-reported outcome set

The German version of the domains assessed by the ICHOMS-SSS were available through <https://www.healthmeasures.net/explore-measurement-systems/promis/intro-to-promis/available-translations>. Please see the homepage for other available translations.

The German version of the PHQ-4 is available through

<https://www.uke.de/dateien/institute/institut-und-poliklinik-f%C3%BCr-psychosomatische-medizin-und-psychotherapie/downloads/gesundheitsfragebogen-phq-4.pdf> (retrieved May 20th, 2021)

Reference: Kroenke, K., Spitzer, R. L., Williams, J. B. W., & Löwe, B. (2009). An ultra-brief screening

scale for anxiety and depression: The PHQ–4. *Psychosomatics*, *50*(6), 613–621. https://doi.org/10.1016/S0033-3182(09)70864-3


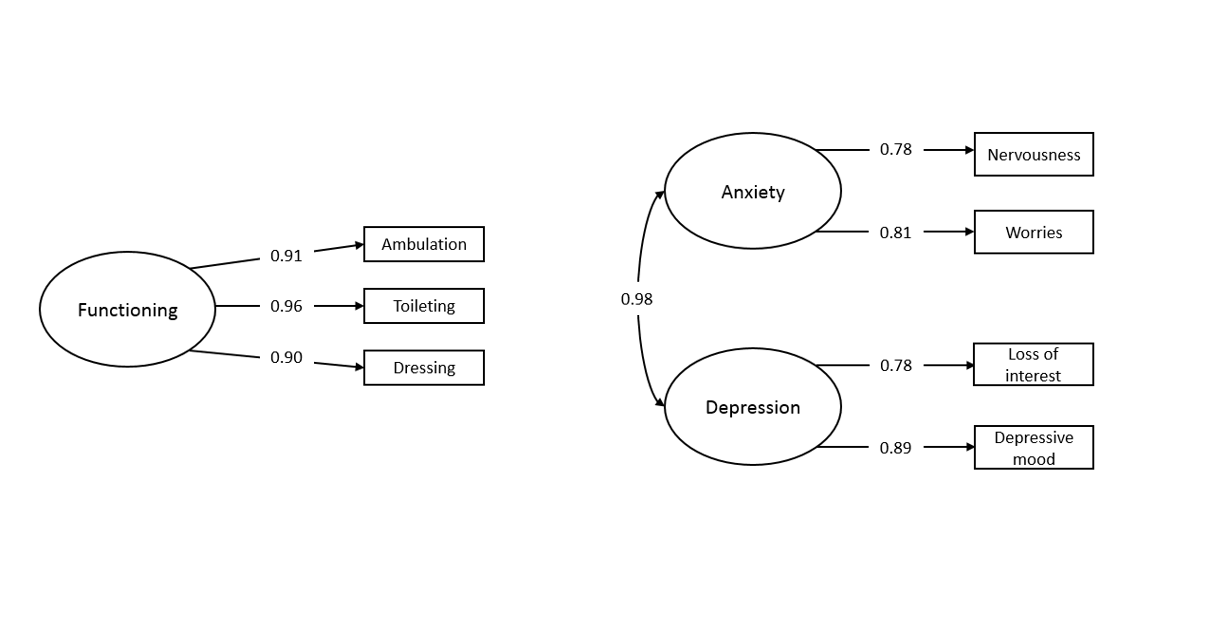


*Figure S1.* Standardized parameter estimates for the single-factor functioning model and the two-factor model of the PHQ-4, both without error terms.

Table S1

*Item and scale characteristics of the functioning scale*

|  | | Item characteristics | | |  |
| --- | --- | --- | --- | --- | --- |
| Item | Cronbach’s α | n | % | Corrected  item-total correlation |  |
| **Functioning** | 0.73 |  |  |  |  |
| Ambulation | | 486 |  | 0.59 |  |
| Without help from another person, with or without device | | 472 | 97 |  |  |
| With help from another person | | 10 | 2 |  |  |
| Unable to walk | | 4 | 1 |  |  |
| Toileting | | 486 |  | 0.61 |  |
| Without help | | | 476 | 98 |  |
| With help | | | 10 | 2 |  |
| Dressing | | 486 |  | 0.55 |  |
| Without help | | 467 | 96 |  |  |
| With help | | 19 | 4 |  |  |

Table S2

*Item and scale characteristics of the PHQ-4*

|  | | Item characteristics | | | | | | |
| --- | --- | --- | --- | --- | --- | --- | --- | --- |
| Item | Cronbach’s α | n | Mean | Standard deviation | Standardized  difficulty | Corrected  item-total correlation | Skewness | Kurtosis |
| **Anxiety** | 0.77 |  |  |  |  |  |  |  |
| Nervousness | | 480 | 0.66 | 0.76 | 0.22 | 0.63 | 1.12 | 1.04 |
| Worries | | 480 | 0.47 | 0.70 | 0.16 | 0.63 | 1.57 | 2.36 |
| **Depression** | 0.80 |  |  |  |  |  |  |  |
| Loss of interest | | 486 | 0.69 | 0.73 | 0.23 | 0.67 | 0.93 | 0.67 |
| Depressive mood | | 481 | 0.54 | 0.69 | 0.18 | 0.67 | 1.28 | 1.69 |
